# Self-assembled quasi-hexagonal arrays of gold nanoparticles with small gaps for surface-enhanced Raman spectroscopy

**DOI:** 10.3762/bjnano.9.188

**Published:** 2018-07-12

**Authors:** Emre Gürdal, Simon Dickreuter, Fatima Noureddine, Pascal Bieschke, Dieter P Kern, Monika Fleischer

**Affiliations:** 1 Institute for Applied Physics, Eberhard Karls University of Tübingen, Auf der Morgenstelle 10, 72076 Tübingen, Germany; 2 Center for Light-Matter-Interaction, Sensors and Analytics (LISA+), Eberhard Karls University of Tübingen, Auf der Morgenstelle 15, 72076 Tübingen, Germany

**Keywords:** block copolymer, electroless deposition, gold nanoparticles, micelle lithography, optical antenna, self-assembly, SERS

## Abstract

The fabrication and optical characterization of self-assembled arrangements of rough gold nanoparticles with a high area coverage and narrow gaps for surface-enhanced Raman spectroscopy (SERS) are reported. A combination of micellar nanolithography and electroless deposition (ED) enables the tuning of the spacing and size of the noble metal nanoparticles. Long-range ordered quasi-hexagonal arrays of gold nanoparticles on silicon substrates with a variation of the particle sizes from about 20 nm to 120 nm are demonstrated. By increasing the particle sizes for the homogeneously spaced particles, a large number of narrow gaps is created, which together with the rough surface of the particles induces a high density of intense hotspots. This makes the surfaces interesting for future applications in near-field-enhanced bio-analytics of molecules. SERS was demonstrated by measuring Raman spectra of 4-MBA on the gold nanoparticles. It was verified that a smaller inter-particle distance leads to an increased SERS signal.

## Introduction

Over the last decades self-assembled layers of gold nanoparticles have taken an important role in emerging nanotechnologies. Noble metal nanoparticles show localized surface plasmon polariton resonances (LSPRs) in the visible and infrared spectral range and exhibit a very strong near-field in their close vicinity [1]. The plasmonic resonances of gold nanoparticles can be varied by changes in size, shape and geometrical arrangement [2–3]. A high density of intense hotspots can be induced by narrow gap sizes and rough surfaces [4–5]. These remarkable optical properties make them attractive for applications in biosensing, biomedical science and as optical antennas [6–8]. In particular, metal nanoparticles can be employed to strongly enhance the signal intensity in chemically specific Raman sensing [9]. This technique is known as surface enhanced Raman spectroscopy (SERS) [10]. Ordered arrays of such particles can be fabricated by different methods. Electron-beam lithography for example is a top-down method which provides good control, but is time consuming and costly. In contrast, the self-assembly of block-copolymers is a bottom-up method, which enables the parallel processing of large areas. A cost-effective photochemical method is block copolymer micelle lithography (BCML), which can be used to create templates on the surfaces of substrates [10–12]. To use the templates for further patterning of the substrate with nanodots, different techniques such as reactive ion etching, thermal evaporation and atomic layer deposition can be used in combination with BCML [13–15]. Here it is important to choose the optimum chain length of the diblock copolymers for obtaining the desired inter-particle spacing [16–17]. It is thus feasible to obtain quasi-hexagonally ordered regular arrays of gold nanoparticles over large areas by simple means. For the fabrication of gold nanoparticles gold salts can be used to load the micelle core, and the copolymer can be removed afterwards with an oxygen plasma treatment [18–20]. For tuning the size of the gold nanoparticles, a combination of micellar nanolithography and subsequent electroless deposition (ED) makes it possible to increase the size of the particles [18]. In this work, we follow the cost-effective and simple photochemical method outlined in [18], but in the present case pursue the goal to fabricate dense ordered arrays of gold nanoparticles with sizes up to >100 nm and single digit gaps on silicon. We first describe the synthesis of gold nanoparticles, which is based on micellar lithography. For tuning the size of the gold nanoparticles we use electroless deposition for different durations. Rough particles with sizes up to 120 nm in diameter are achieved in quasi-hexagonally ordered arrays, resulting in a high density of hotspots as has been shown for similar raspberry-like nanostructures [21–22].

Next, the optical properties of the samples are characterized by measuring the scattering spectra of selected gold nanoparticles. Finally, we demonstrate SERS enhancement by measuring Raman spectra of 4-mercaptobenzoic acid (4-MBA) molecules that are adsorbed to the gold nanoparticles.

## Experimental

### Block-copolymer micellar lithography

1 × 1 cm^2^ silicon substrates were cleaned with acetone in an ultrasonic bath for two minutes. Then they were rinsed with isopropanol, and finally dried with nitrogen gas. A symmetric diblock copolymer (polystyrene-block-poly-2-vinylpyridine, PS(133000)-block-P2VP(132000), polymer source) was dissolved in toluene at a concentration of 1 mg/mL and stirred for 2 days. The micelles were loaded with chlorauric acid (HAuCl_4_, loading parameter (*L* = 0.5), Sigma-Aldrich) and stirred again for 2 days. Spin-coating was applied to cover the substrate with a monolayer of the gold-loaded micelles (30 s at 2000 rpm).

### Electroless deposition

A quartz glass slide was placed on top of the substrate after a drop of about 10 µL of water was applied. The assembly was then exposed for 4 min to deep UV light (254 nm, 85 W). After this step, the substrate was placed in an aqueous solution of enthanolamine (2 mM, Sigma-Aldrich) and potassium gold(III) chloride (KAuCl_4_, 0.1 wt %, Sigma-Aldrich), to grow the gold precursor particles with the electroless deposition process. Reactive ion etching (Oxford Plasmalab 80 Plus) was used to remove the polymer with an oxygen plasma treatment with the following settings: process pressure 100 mTorr, power 100 W, temperature 20 °C and duration of the etching process 60 s. To measure the inter-particle spacing and sizes of the gold nanoparticles in this work we used a Scanning Electron Microscope (SEM) (Hitachi SU 8030).

### Darkfield spectroscopy

The scattering spectra of gold nanoparticles were measured with a custom-built dark-field spectroscopy setup. A 50× objective (Mitutoyo BD Plan APO SL 50X) was used for imaging and taking the spectra. The samples were illuminated by a laser driven light source (Energetiq EQ-99-FC) at an incident angle of light of about 45°. The spectra were taken with an Andor Shamrock SR-303i spectrometer equipped with an iDus DU416A-LDC-DD detector.

### Raman spectroscopy

The gold nanoparticles were incubated for 22 h with a 5 mM solution of 4-MBA (Sigma Aldrich) in ethanol. After this process, the substrate was rinsed with ethanol and dried with nitrogen gas. The Raman spectra were measured in a confocal Raman spectrometer (LabRam HR 800, Horia JobinYvon) using a 632.8 nm He–Ne-laser with a laser power of 50 mW and a 50× objective. The laser aperture was set to 1000 µm, the slit size to 200 µm and the grating had 1800 lines/mm, resulting in a spectral resolution of ≈2 cm^−1^. For all measurements the exposure time was set to 60 s to reduce noise.

## Results and Discussion

We use the bottom-up method of BCML combined with ED to fabricate tunable gold nanoparticles forming quasi-hexagonal arrays on a silicon substrate. The optical properties of the gold nanoparticles are investigated by dark-field spectroscopy. Finally we show that by tuning the size (and thus the inter-particle spacing) of the particles, a higher SERS signal intensity could be obtained.

**Figure 1:**
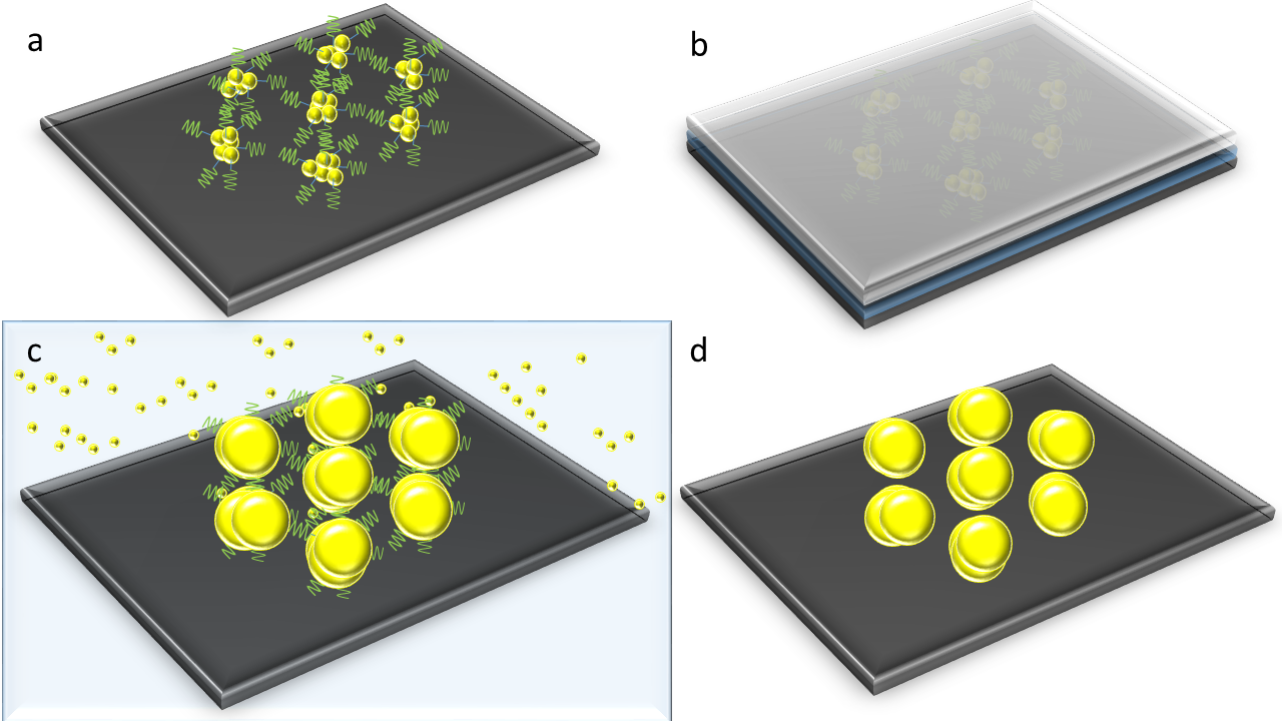
Schematic illustration of the preparation of variable-size gold nanoparticle arrays on top of a silicon substrate: (a) Gold nanoparticles with block copolymer micelles after spin-coating. (b) Deep UV illumination with water and a quartz glass on top of the substrate. (c) Electroless deposition. (d) Substrate after oxygen plasma, which removes the organic components.

The PS-b-P2VP diblock copolymer is dissolved in toluene, which is an apolar solvent. An apolar solvent dissolves preferentially the PS block [23]. The hydrophobic PS forms the shell, and the hydrophilic P2VP the core of the spherically shaped micelles [24]. Within their core gold salt can be assembled, which is bonded by protonization or complexation [25]. The loaded spherical micelles form a hexagonal array when being deposited on a substrate. Exposing them to an aqueous environment promotes a morphological change of the spherical micelles [18]. In the next step, the micelles are treated with UV irradiation, which causes the gold salt particles in the center to grow bigger by photochemical growth [18]. To enlarge the metal precursor particles even further in a controlled fashion, an electroless deposition step using potassium gold(III) chloride was performed [18,26]. To reduce the gold ions to elemental gold, a solution of ethanolamine as a reducing agent can be used [18]. The final size of the gold particles can be tuned by the duration of the process [18]. A schematic overview of the fabrication process is shown in Figure 1. In a first step a silicon substrate is coated with gold-loaded polymer micelles (Figure 1a) via spin-coating. In a second step the micelles are exposed to deep UV illumination while the substrate is covered with a quartz glass slide (Figure 1b). In a third step the nanoparticles are enlarged by electroless deposition (Figure 1c), and finally the polymer is removed by an oxygen plasma treatment (Figure 1d).

**Figure 2:**
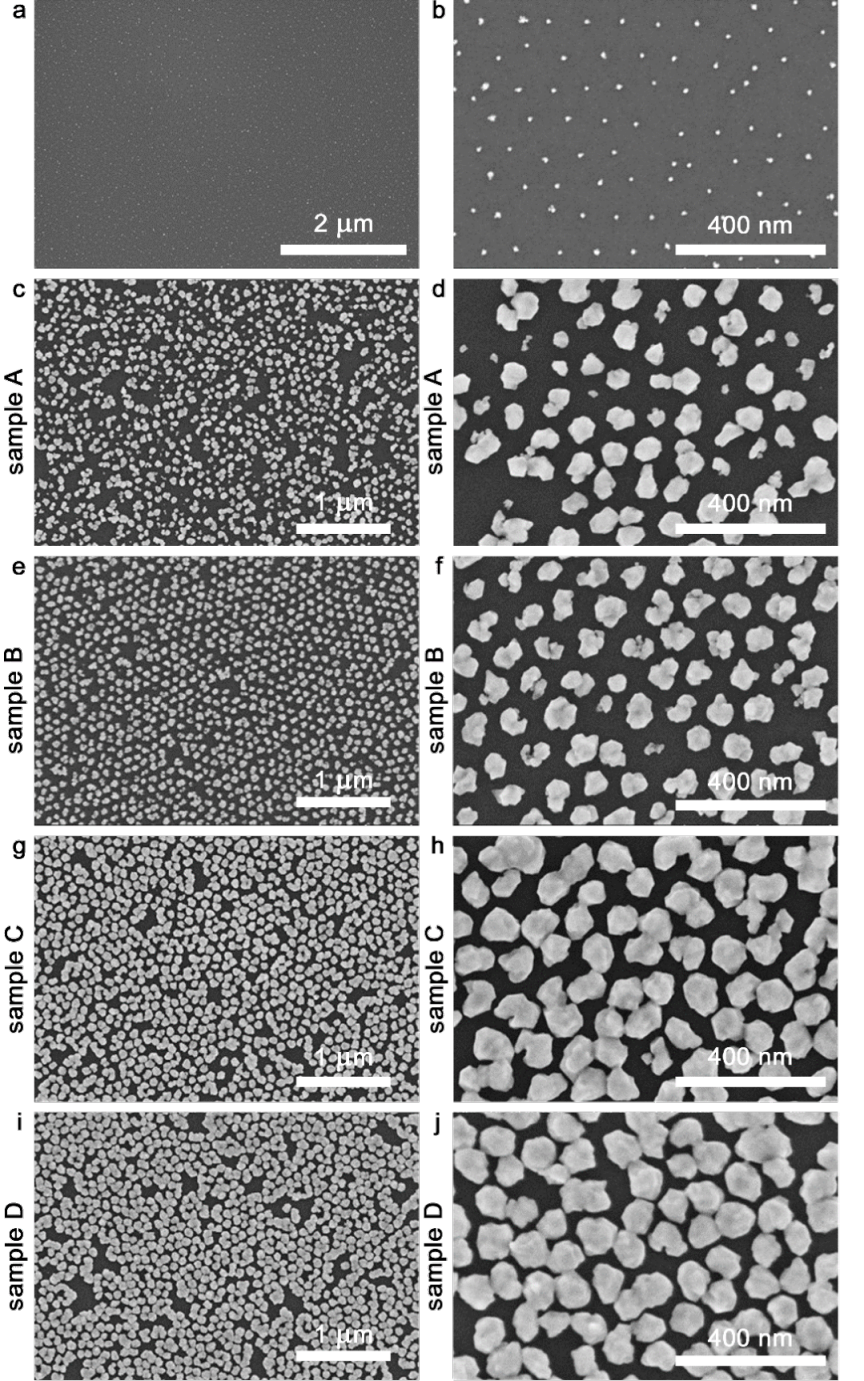
SEM images of the gold precursor particles after oxygen plasma treatment (a, b) and gold nanoparticles after electroless deposition and oxygen plasma treatment: (c, d) sample A with 30 min ED, (e, f) sample B with 30 min ED, (g, h) sample C with 90 min ED, (i, j) sample D with 90 min ED. Scale bars: (a) 2 µm; (c, e, g, i) 1 µm; (b, d, f, h, j) 400 nm.

SEM images of the primary distribution of the gold precursor particles without any size increase by ED confirming that the micelles cover the entire silicon surface are shown in Figure 2a,b. The distribution is mostly regular, except for occasional defects, and shows a roughly hexagonal order. The center-to-center-spacing of the ordered particles amounts to 109 ± 20 nm. After deep UV illumination, electroless deposition and oxygen plasma treatment, SEM images are taken at two different magnifications, which are shown in Figure 2c–j. For a direct comparison between SERS platforms with large and small gaps, four substrates were fabricated, two each with identical parameters for process assessment.

In Figure 2c,d and 2e,f, representative images of gold nanoparticles after an electroless deposition step of 30 min are shown. The first substrate (A) (Figure 2c,d) exhibits an average nanoparticle size (nps) of 66 ± 25 nm and an average inter-particle distance from edge to edge (ipd) of 56 ± 9 nm. The second substrate (B) (Figure 2e,f) shows an nps of 73 ± 16 nm and an ipd of 33 ± 6 nm. Substrate A shows a lower degree of order than B. Two more samples were prepared with the same process steps, but with an electroless deposition of 90 min instead of 30 min. In Figure 2g,h sample C has an nps of 96 ± 12 nm and an ipd of 17 ± 6 nm. The second sample (D) in Figure 2i,j shows an nps of 97 ± 10 nm and an ipd of 14 ± 9 nm. The statistical ipd of 14 ± 9 nm indicates the presence of a considerable number of sub-10 nm gaps. Comparing the particle sizes, one finds a significant variation between the 30 min samples, while the 90 min samples exhibit very similar arrangements. The results are summarized in Table 1. The inter-particle distances were measured directly from the SEM images, and averaged over ten measurements. The nanoparticle sizes for the samples A and B were evaluated by using the method described in the next paragraph. Because many of the particles in samples C and D touch each other, they could not be separately discerned by this method, and their nps had to be measured manually from the SEM images, also averaging over ten measurements.

**Table 1:** Measured average particle sizes and interparticle distances for the different samples.

Sample	ED duration	Avg. nanoparticle size	Avg. inter-particle distance

A	30 min	66 ± 25 nm	56 ± 9 nm
B	30 min	73 ± 16 nm	33 ± 6 nm
C	90 min	96 ± 12 nm	17 ± 6 nm
D	90 min	97 ± 10 nm	14 ± 9 nm

**Figure 3:**
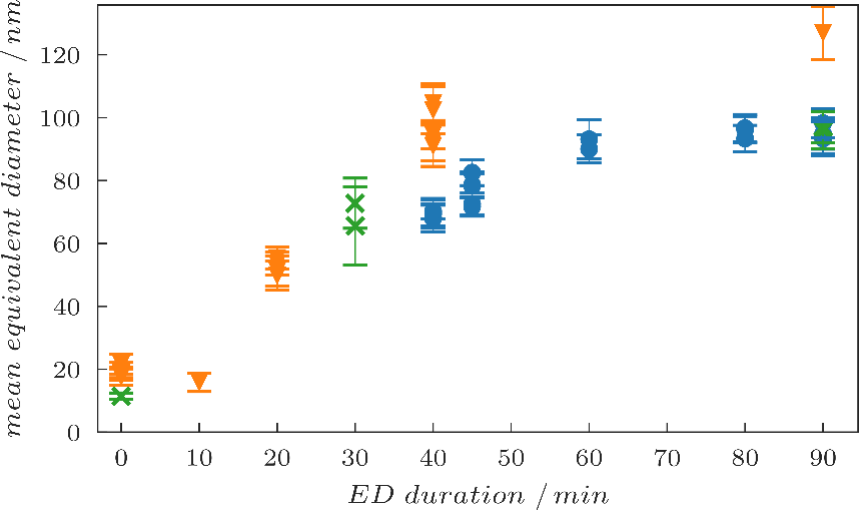
Dependence of the mean equivalent diameter of the gold particles on the ED duration. Three separate sets of samples were fabricated and evaluated. The series are color coded as orange triangles, blue circles and green crosses. Each marker represents the data from one SEM image. The error bars indicate the deviations of the mean equivalent diameter within the respective SEM images. The green crosses correspond to the samples that are shown in Figure 2. The values for the green crosses for an ED duration of 90 min were evaluated manually from the SEM images, all others were calculated by using the method described in the main text.

In order to find the dependence of the gold particle diameter on the ED time, two additional series of samples with different time steps were fabricated. The preparation parameters were similar to the ones shown before, only the polymer concentration was reduced to 0.7 mg/mL and the loading parameter was set to *L* = 1. The results are summarized in Figure 3. The SEM images for each sample were evaluated using a python script that applies a threshold in order to generate binary images. Blob detection is used to find the particles in the binary images and to evaluate the pixel count for each individual particle. From this pixel count, the area coverage and thus the mean equivalent diameter of the particles is calculated, assuming perfectly round particles. A histogram of all the diameters is calculated and a Gaussian is fitted to this histogram. This allows us to extract the mean equivalent diameter as well as the full-width-at-half-maximum (fwhm) of the diameter distribution, which is indicated by the error bars in Figure 3. Since in reality the particles are irregular and exhibit some surface roughness, the equivalent diameters underestimate the maximum outer diameter, and thus the minimum gap sizes to neighbouring particles may be even smaller than indicated by this evaluation. A general trend of increasing particle diameters with increasing ED times can be observed. The growth goes into saturation when the particle size approaches the interparticle spacing. Before ED, the gold-loaded micelles start with sizes around 10 nm to 20 nm. As the ED duration increases, their size increases up to about 100 nm to 120 nm. A systematic offset can be discerned between the separate test series, indicating that the process is highly sensitive to the exact preparation conditions during the fabrication process even when the same recipe is followed. In addition, the center-to-center spacing varies slightly from sample to sample.

**Figure 4:**
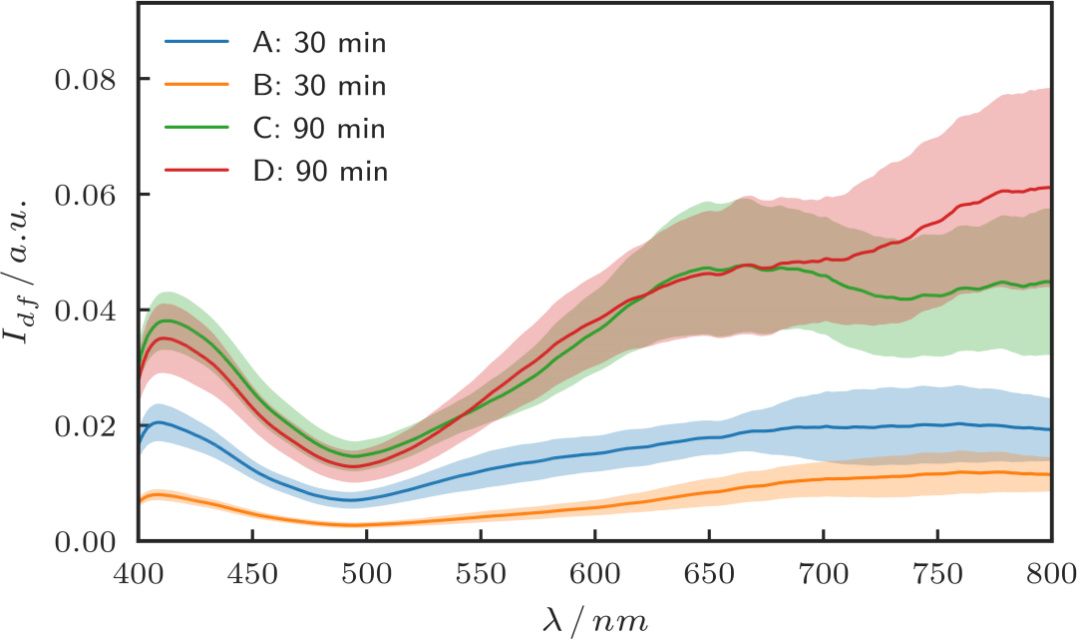
Mean dark-field spectra of the different samples. For each sample 25 spectra at different positions were taken and averaged. The shaded regions show the standard deviation of the average.

To compare the SERS signal of smaller particles with larger gaps to that of larger particles with small gaps, the optical properties of the samples shown in Figure 2 were further analyzed using dark-field spectroscopy. For every sample, 25 measurements at different points were taken and averaged. The results are shown in Figure 4. The bigger particles (sample C and D) show an overall increase in the scattering intensity compared to the smaller ones (A and B), as one would expect for Rayleigh scattering. The curves exhibit very broad spectral features.

To measure the SERS signal, the gold nanoparticles were covered with a self-assembled monolayer of 4-MBA. Because the thiol-group of the 4-MBA molecules has a very high affinity for gold [27], and the samples were rinsed thoroughly with ethanol to remove any unbound molecules, we can assume that mostly 4-MBA molecules are present on the gold surfaces and not on the substrate.

**Figure 5:**
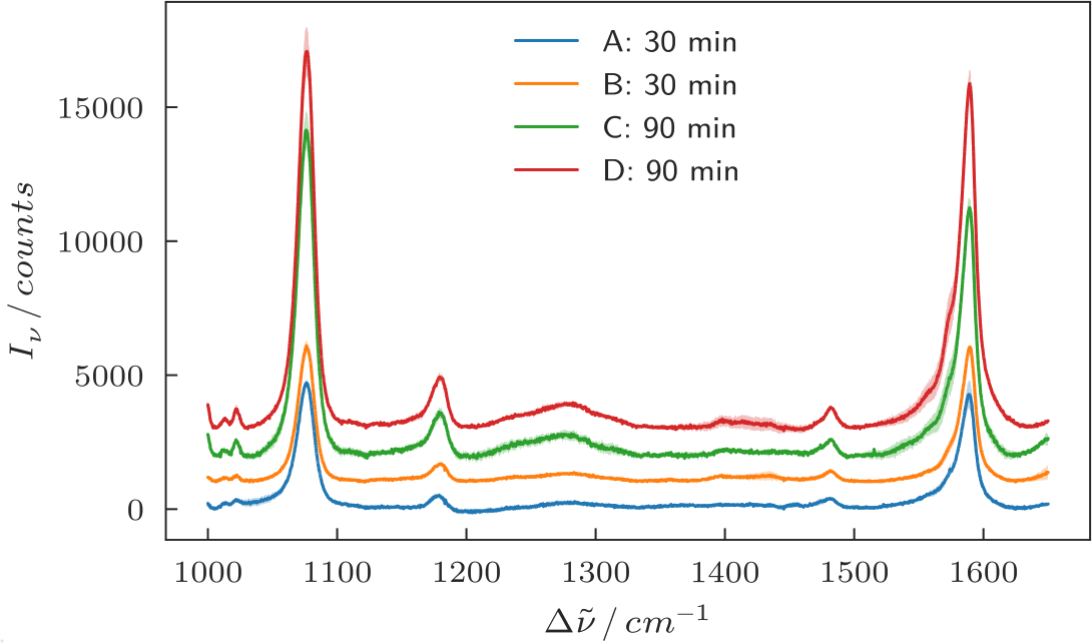
Background-corrected mean Raman spectra of the four different samples. For each sample three Raman spectra at three random positions on the samples were taken and averaged. The shaded regions show the standard deviation of the average. The spectra were offset vertically for clarity.

Raman spectra were recorded as described above at three different positions on every sample and averaged. For the excitation the laser wavelength of 632.8 nm was chosen, since according to Figure 4 it appears to have good spectral overlap with the plasmon resonances (maxima in the scattering intensity) of the larger gold particles, and is thus expected to excite strong hotspots in the gaps. The intensity of the characteristic Raman bands for 4-MBA at 1085 cm^−1^ and 1590 cm^−1^ were evaluated [28]. The background-corrected peak intensities are summarized in Table 2, denoted as “raw”. By looking at the SEM images in Figure 2, it is obvious that the samples show a difference in the amount of gold that is present, which also means that for each sample a different amount of molecules attached to gold is present in the focal spot of the Raman laser. To approximately correct for the different amounts of molecules on the different samples one can use the filling factor (area coverage) of the samples: A threshold was applied to the SEM images, and the white pixels representing the presence of gold were counted. The filling factor was then calculated by dividing the white pixel count by the number of pixels of the image. This represents a measure for the average particle size as well as for the density of the particles, and correspondingly it also provides a measure for the number of molecules on gold per unit area. The Raman intensities were then divided by this filling factor, which results in filling factor-corrected intensities. The resulting filling factors and corrected Raman intensities (denoted as “corrected”) are shown in Table 2, while the raw background-corrected Raman intensities as measured are visualized in Figure 5.

**Table 2:** Filling factor and measured Raman intensities (raw: background corrected raw data, corrected: Raw spectra normalized by filling factor) for all samples.

Sample	Filling factor	Raman int. at 1085 cm^−1^ [k counts]		Raman int. at 1590 cm^−1^ [k counts]	
		raw	corrected	raw	corrected

A	0.31	3.8 ± 0.1	12.2 ± 0.3	3.1 ± 0.4	10.1 ± 1.3
B	0.33	4.3 ± 0.2	13.1 ± 0.6	3.8 ± 0.1	11.5 ± 0.4
C	0.56	10.3 ± 0.5	18.6 ± 1.0	6.8 ± 0.2	12.2 ± 0.4
D	0.62	12.0 ± 0.8	19.5 ± 1.3	9.3 ± 0.3	15.0 ± 0.5

**Figure 6:**
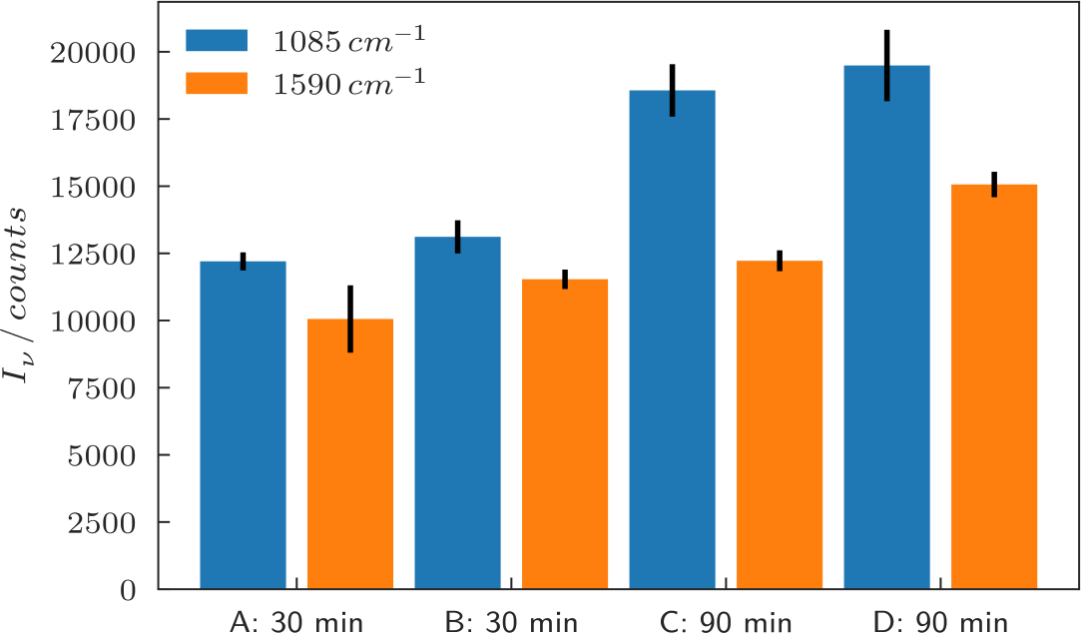
Filling factor-corrected Raman intensities of the different samples, for the two peaks at 1085 cm^−1^ and 1590 cm^−1^. The average of 3 measurements for each sample is shown, the black bars denote the standard deviation.

By looking at the raw Raman spectra for the different samples one can see that the larger particles show higher Raman intensities than the smaller particles by more than a factor of 2. Of course, in this case the larger gold surface and thus the higher number of molecules was not taken into account. If the Raman intensities are corrected for the filling factor as explained above, the difference between the samples becomes smaller, but still the larger particles show an increased Raman signal, particularly for the peak at 1085 cm^−1^. Figure 6 shows a comparison of the corrected Raman intensities for the different samples where this increase is clearly visible. This effect may be explained by the much shorter mean inter-particle distances between the larger nanoparticles, including some very narrow gaps due to the statistical variation, which causes an increased coupling between the particles and thus an increased near-field [29].

**Figure 7:**
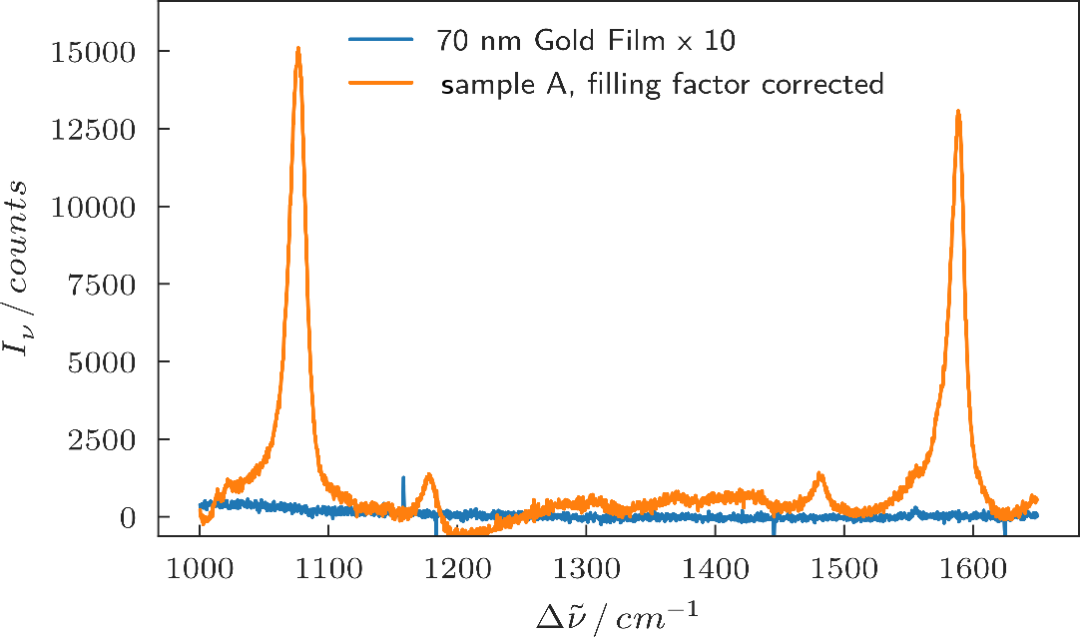
Filling factor-corrected Raman spectrum of sample A compared to a Raman spectrum on smooth gold film.

To estimate a lower boundary for the enhancement factor of the particles we compared the corrected Raman spectra of sample A to a measurement of 4-MBA on a smooth gold film with a thickness of 70 nm, also on a silicon substrate. Both samples were treated in exactly the same way. The spectra are shown in Figure 7. For the gold film no signal was observed, and thus we assume that the upper limit of the signal is the peak-to-peak noise in the measurement. By dividing the maximum corrected signal of the Raman mode at 1085 cm^−1^ by the peak-to-peak noise of the measurement on the gold film we obtain a lower limit of the enhancement factor of ≈300. This is a very conservative lower limit, and compared to values commonly reported in literature it is significantly smaller, but we would like to stress that the estimation of SERS enhancement factors is inherently difficult and is still a much discussed topic within the community [30–31].

As can be seen in the SEM images for the samples with 90 min ED, the particles show average separation distances around 15 nm and individual separations down to only a few nanometers. This means that the method presented here allows for the fabrication of nano-particles that exhibit very small mode volumes and high near-fields. The fabrication is based on bottom-up processes and thus offers the possibility to scale it up to bigger substrates and higher throughput. The high near-fields and the ease of fabrication make these structures particularly suitable for sensing applications, for example for SERS as it was shown here.

## Conclusion

In conclusion, we describe a cost-effective, scalable, parallel method for the fabrication of quasi-hexagonally ordered arrays of nanoparticles with particle sizes up to 120 nm and gap sizes down to few nanometers, which are fabricated by block copolymer micellar nanolithography combined with electroless deposition. The resulting particle arrangements are compared for samples prepared with 30 min vs 90 min ED. The dark-field scattering intensity is compared for the different nanoparticle sizes. We demonstrate the SERS effect exhibited by these samples by measuring Raman spectra of 4-MBA that is adsorbed to the gold nanoparticles. The spectra show an increase in Raman intensity for larger particles and smaller gap sizes by a factor of >2. The surfaces with the narrower gap sizes result in higher intensities even when correcting for the different particle sizes and area coverage. This effect may be attributed to a stronger near-field coupling between the particles due to smaller inter-particle distances.
